# The day-of-week (DOW) effect on liberalism-conservatism: Evidence from a large-scale online survey in China

**DOI:** 10.3389/fpsyg.2022.1074334

**Published:** 2023-02-14

**Authors:** Shengquan Ye, Justin Juk Man So, Ting Kin Ng, Mac Zewei Ma

**Affiliations:** ^1^Department of Social and Behavioural Science, Colleague of Liberal Arts and Social Sciences, City University of Hong Kong, Kowloon, Hong Kong SAR, China; ^2^Institute of Epidemiology and Health Care, University College London, London, United Kingdom; ^3^Department of Psychology, Lingnan University, Tuen Mun, Hong Kong SAR, China

**Keywords:** day-of-week effect, liberalism-conservatism, large-scale survey, affective states, cognitive states

## Abstract

**Introduction:**

Past research has shown that psychological states tend to fluctuate across the days of a week, which is referred to as the day-of-week (DOW) effect. This study investigated the DOW effect on liberalism-conservatism among Chinese people by testing two competing hypotheses. According to the cognitive states hypothesis, it was predicted that liberalism would be high on Mondays but gradually decrease to Fridays due to the depletion of cognitive resources over the weekdays. In contrast, the affective states hypothesis predicted the opposite, considering the more positive affect brought by the approaching weekends. Both hypotheses predicted the level of liberalism would peak at weekends.

**Methods:**

Data (*n* = 171,830) were collected via an online questionnaire, the Chinese Political Compass (CPC) survey, which includes 50 items to measure people’ liberalism-conservatism in three domains (i.e., political, economic, and social).

**Results:**

The results showed the level of liberalism decreased gradually from Mondays until Wednesdays, rebounded from Wednesdays to Fridays, and peaked at weekends.

**Discussion:**

The V-shaped pattern suggested that the DOW fluctuation in liberalism-conservatism could derive from the synergy of both cognitive and affective processes, instead of either one alone. The findings have important implications for practice and policy-making, including the recent pilot scheme of 4-day workweek.

## Introduction

Human activities are often organized in a weekly cycle. Most people work or study on the five weekdays and rest on the two weekend days. Consistent with this regular pattern, research has shown that people assign different meanings to the days of a week (Ellis et al., 2015), such as the so-called blue Monday (first working day of a week) and happy Friday (last working day of a week). Numerous health and well-being outcomes have been found to vary across the days of a week (Barnett and Dobson, 2005; Ellis and Jenkins, 2012; Helliwell and Wang, 2014), which is generally referred to as the day-of-week (DOW) effect. Recent studies also found that the DOW effect on individual behaviors could have society-wide implications. For example, the experimental study by Sanders and Jenkins (2016) found that participants were more risk-averse on Thursdays than other weekdays. They further examined the actual daily polls of the Scottish Independence Referendum and the United Kingdom European Union membership referendum and suggested that elections traditionally being held on Thursdays may be systematically biased towards the direction of risk-averse.

This study aims to contribute to the current DOW literature in three ways. First, although past research has identified a significant DOW effect on voting behavior (Sanders and Jenkins, 2016), voting *per se* is a complex process that can be influenced by multiple factors including the beliefs that people are holding (Leimgruber, 2011). Therefore, the present study was focused on the DOW effect on liberalism-conservatism, which is important predictors of the actual voting behavior (Morgan and Wisneski, 2017). Liberalism-conservatism, which involves one’s social and political attitudes, serves as a schema for people to process information related to politics (Morgan and Wisneski, 2017). Therefore, investigating the DOW effect on liberalism-conservatism contributes to a more comprehensive understanding of voting behavior and other psychological outcomes such as well-being, which was found to vary from weekdays to weekends (Ryan et al., 2010). Second, research on the DOW effect has primarily been conducted in the West. Since ideology is culture-specific, it is necessary to investigate this effect in Eastern cultures such as China, which has been economically and politically different from the West. Pan and Xu (2018) found that Chinese ideology encompassed three domains: political, economic, and social. As these three domains are highly correlated and reflect the liberalism-conservatism ideology (Huang et al., 2019), they can be combined into a single dimension of Chinese ideology. For example, people who were conservative in the economic domain (e.g., support for planned economy) also tended more to support conservatism in the political and social domains (Huang et al., 2019). Third, the current study utilized data from a large-scale online survey to investigate the DOW effect on liberalism-conservatism. By employing big data in behavioral science (Kosinski et al., 2016), researchers can detect patterns of behavior with greater statistical power that might otherwise be missed or misinterpreted due to factors like smaller effect sizes and greater sample variability (Qiu et al., 2018). Unlike the studies where experimental data and controlled protocols were employed, the data from the real-world settings could provide findings with higher ecological validity. This could be particularly beneficial as many organizations in Europe, as well as in North America and New Zealand, have been or will be piloting the 4-Day Week Global, where employees are required to work for only 4 days instead of five, while maintaining the same level of productivity. Understanding the DOW effect on psychological processes including ideological thinking have significant practical implications for the new 4-day workweek arrangement.

### Liberal vs. conservative ideology

The construct of ideology is often studied using the unidimensional continuum of liberalism versus conservatism (e.g., Nail et al., 2009). Liberals are likely to favor equality (receiving equal outcomes regardless of contributions), whereas conservatives tend to value equity (receiving outcomes proportional to contributions; Schlenker et al., 2012). Concerning attitudes toward societal changes, while liberals tend to approve progressive changes (accepting recognized social trends), conservatives tend to approve reactionary changes (reversal of social trends and return to tradition; Proch et al., 2019). The former is positively related to openness and curiosity, while the latter is related to preserving conventions and order (Carney et al., 2008). Past research has shown liberalism-conservatism can influence human decision-making. For instance, liberals are more supportive for organ donation (Chan, 2019) and more willing to take action to address the issue of climate change (McCright et al., 2016) than conservatives. Furthermore, liberalism-conservatism can vary to some extent under certain conditions. For instance, a meta-analysis showed that threatening situations predicted political conservatism as people became resistant to change and tended to justify inequalities in order to manage uncertainty and defend against outside threat (Jost et al., 2003). It was also found that experimentally induced threats led the liberals to hold more similar views as the conservatives regarding topics such as homosexuality and abortion, providing support that liberalism-conservatism was changeable across social situations (Nail et al., 2009). Similarly, Bryan et al. (2009) found that students primed to think of their success as a result of help from others (liberalism-oriented) showed more support for liberalism, suggesting that liberalism-conservatism could be influenced by the comparative salience of different schematic activations under different circumstances. With the previous findings, it would be worthwhile to investigate whether liberalism-conservatism changes in response to different life situations across the 7 days of the week. Below we present two theoretical accounts regarding how this may take place and propose the respective hypotheses.

### DOW effect: Affective vs. cognitive states hypotheses

First, the DOW effect could influence liberalism-conservatism through influencing affective states. According to the whole trait theory (Fleeson and Jayawickreme, 2015), behaviors are not only influenced by relatively stable traits, but also the momentary states and situational factors. Wilson et al. (2017) found that positive state affect predicted state openness, which is a strong predictor of liberalism (Carney et al., 2008). Hence, the DOW effect on liberalism-conservatism can be reasoned according to the affective state changes during the week. Research has showed that people’s affect fluctuates across the 7 days of the week. Specifically, people experience more negative affect from Mondays to Thursdays (Taylor, 2006; Stone et al., 2012), especially on Mondays (Ellis et al., 2015). On the contrary, Fridays and the weekend days were strongly associated with positive affect (Ryan et al., 2010). These patterns have also been supported by neuropsychological studies. For example, Schlotz et al. (2004) found that chronically stressed individuals had higher levels of stress, as indicated by cortisol awakening responses, during the weekdays but not weekends. According to the different affective states people experience across different days of the week, the following hypotheses regarding the DOW effect on liberalism-conservatism are proposed. First, liberalism would peak near weekend days because of more positive affective states. Second, liberalism would drop to the lowest level on Mondays due to negative affective states. Third, liberalism would increase steadily in the following weekdays until it peaks again on the weekends.

**Figure 1:**
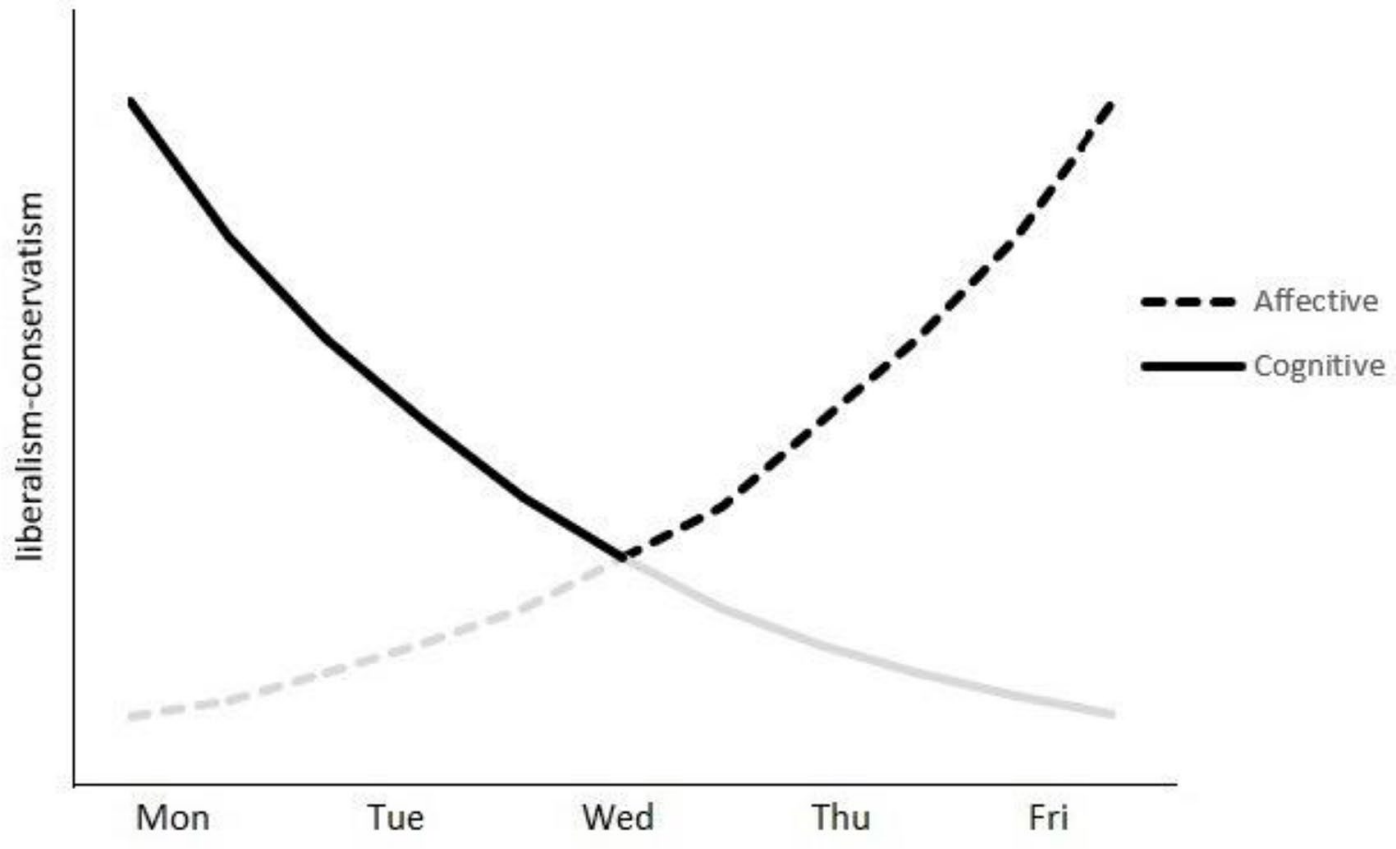
The cognitive and affective states hypotheses. Note. Weekends are not included as both cognitive and affective states hypotheses suggest the highest levels of liberalism-conservatism. The dark lines illustrated the predominant effects.

Second, the fluctuations in liberalism-conservatism may be explained by changes in cognitive states across different days of the week (Buchel and Dolan, 2000; LeDoux, 2000; Charpentier et al., 2016). According to the occupational stress literature, employees experience depletion of physical and cognitive resources during work days and recovery of energy during holidays (Weigelt et al., 2021). Weigelt et al. (2021) found that employees’ energy increased during weekends and decreased continuously across weekdays. In recent decades researchers have elaborated the cognitive-affective interactions model, which posits that humans’ decision-making is the equilibrium of the higher-order cognition and affective states. The higher-order cognition is also highly related to ideological thinking. Several studies have reported that openness to experience, a personality trait highly related to liberalism (Alford and Hibbing, 2007; Carney et al., 2008; Mondak and Halperin, 2008). Cognitive functioning, including cognitive flexibility and openness to novelty (Soubelet and Salthouse, 2010), has been found positively associated with liberalism and negatively related to conservativism (Jost et al., 2003; Carney et al., 2008). Therefore, the fluctuating cognitive state during the week offers an alternative explanation for the fluctuations in liberalism-conservatism from Mondays to Sundays. The effort-recovery theory (Meijman and Mulder, 1998) postulates that people need off-job time to recover and replenish their mental resources, which would produce short-term benefits at the beginning of the working week. In fact, a longitudinal study found that employees reported better task performance on Monday (right after the weekend) than on Tuesdays (Fritz and Sonnentag, 2005). Hence, as people generally rest on weekends, we hypothesized that liberalism would peak on the weekend days due to better cognitive resources and state. Starting on Mondays, liberalism would decrease gradually throughout the weekdays when cognitive resources continue to deplete. Figure 1 illustrates the predicted weekly patterns of liberalism-conservatism according to the two theoretical accounts and their respective hypotheses.

## Materials and Methods

### Sample and procedure

This study adopted the 2014 Chinese Political Compass (CPC) dataset, a well-established and large online survey available on the Internet.^1^ There were 171,830 respondents (male: 110,110, female: 61,545, unreported: 175), with an average age of 24.11 (*SD* = 7.43). Since this survey was set up at Peking University in 2007, it had gained increasing attention and been completed by a very large sample of internet users voluntarily. This study only uses the 2014 data, which is available to the public. As pointed out by Pan and Xu (2018), there are both pros and cons of this opt-in sample. On one hand, it provided valuable data on questions that may not appear in approved social surveys. Besides, voluntary participation is often associated with higher motivation to provide accurate answers (Chang and Krosnick, 2009). On the other, the sample tended to bias towards young, male, well-educated participants living in more developed areas of China. The dataset recorded the exact time and date of the responses, which were converted to the time zone of Beijing, China. The days of the week were then coded based on the converted dates.

### Measures

The CPC survey consists of 50 items measuring three domains: political (e.g., “People should not have universal suffrage if they have not been educated about democracy”), economic (e.g., “If the price of pork is too high, the government should intervene”) and social (e.g., “Two adults should be free to engage in voluntary sexual behavior regardless of their marital status.”). Each item was rated on a 4-point scale. Pan and Xu (2018) reported that the 50-item CPC represented a unidimensional construct, which is liberalism versus conservatism. In their analysis, five items (i.e., Q12, Q3, Q2, Q6, and Q17) were found to best represent liberalism, while five other items (i.e., Q9, Q46, Q20, Q48, and Q33) were found to best represent conservatism. For parsimony, a liberalism-conservatism score was constructed by deducting the mean of the five conservatism questions from the mean of the five liberalism questions. As a result, higher scores indicated higher levels of liberalism (or lower levels of conservatism). This score was not only highly correlated (*r* = 0.919) with the extracted factor score based on principle component analysis, but offered more convenient interpretation and better comparability due to the retention of the original rating scale.

### Statistical analyses

A one-way analysis of covariance (ANCOVA) was employed to inspect the differences in liberalism-conservativism across the 7 days of the week. Specifically, the dependent variable was the liberalism-conservatism score and the independent variable was the categorical variable indicating the days of a week. Gender, age, income and education level, which were previously found to be associated with liberalism-conservatism (Box-Steffensmeier et al., 2004; Zeng, 2014; Pan and Xu, 2018), were controlled in the analysis. *Post-hoc* analyses with Sidak adjustment were performed to examine the pairwise differences among the 7 days of the week.

## Results

Table 1 presents the descriptive statistics of the liberalism-conservatism score. A one-way ANCOVA indicated that there were significant differences across the 7 days of the week, *F*(6, 170,753) = 60.79, *p* < 0.001, η_p_^2^ = 0.002. The effects of covariates were all significant. People who were male (*p* < 0.001, η_p_^2^ < 0.001), younger (*p* = 0.006, η_p_^2^ < 0.001), and had higher income (*p* < 0.001, η_p_^2^ = 0.006) and education level (*p* < 0.001, η_p_^2^ = 0.001) tended to be more liberal.

**Table 1:** Descriptive statistics of the liberalism-conservatism score.

		Unadjusted		Adjusted	
	*n*	M	SD	M	SE
Monday	34,402	4.95	1.60	4.95^a^	0.01
Tuesday	25,604	4.95	1.67	4.93^ab^	0.01
Wednesday	19,477	4.83	1.68	4.81^c^	0.01
Thursday	20,668	4.84	1.63	4.85^cd^	0.01
Friday	23,959	4.88	1.61	4.89^bd^	0.01
Saturday	24,679	5.03	1.61	5.05^e^	0.01
Sunday	21,975	5.01	1.60	5.02^e^	0.01

Note. Adjusted means are obtained by controlling for gender, age, income, and education. Adjusted means that do not share a common superscript differ significantly at *p* < 0.01 (*Post-hoc* comparisons with Sidak adjustment).

*Post-hoc* comparisons with Sidak adjustment were conducted to further examine the main effect of DOW (see Table 1). As predicted by both affective and cognitive states hypotheses, liberalism peaked during weekends, with no significant difference being detected between Saturdays and Sundays. After weekends, the level of liberalism was significantly lower on Mondays and Tuesdays (*p* < 0.001), which further dropped significantly to the lowest on Wednesdays (*p* < 0.001). The level of liberalism gradually increased afterwards until it peaked on Saturdays.

## Discussion

The present study employed data from a large-scale online survey to examine the weekly patterns of changes in liberalism-conservatism in China. The results demonstrated that the level of liberalism peaked during the weekend days, followed by gradual decrease from Mondays to Wednesday, and then rebounded on Thursday and Friday. This DOW pattern was consistent with the previous findings on voting behavior in the United Kingdom (Sanders and Jenkins, 2016). As the effects were based on large dataset and robust in different sociopolitical contexts, the fluctuation patterns found in the present and previous studies may reveal a pan-cultural phenomenon that deserves close attention, considering the regular weekly cycle adopted worldwide and the implications at both individual and societal levels.

As predicted by both cognitive and affective states hypotheses, the level of liberalism peaked on weekend days. Interestingly, the V-shaped pattern in the level of liberalism may suggest there could be a mixed effect of both cognitive and affective states. According to cognitive states hypotheses, the level of liberalism would decrease from Monday to Friday. On the contrary, the affective states hypotheses predicted the level of liberalism would increase from Monday to Friday. The results did not provide direct support to either of the two predictions. Instead, the V-shaped fluctuation of liberalism seems to result from the aggregation of the two effects by cognitive and affective states, as illustrated in Figure 1. On one hand, during the first half of the weekdays (i.e., from Monday to Wednesday), there could be fast depletion of cognitive resources that lead to substantial decrease in the level of liberalism. The affective states may remain relatively low without large change. On the other hand, during the second half of the weekdays (i.e., from Wednesday to Friday), the cognitive states may have dropped to a low level and become relatively stable, while the affective states gradually become increasingly positive due to the expectation for the approaching weekends. Due to the nonlinear change in the processes, the fluctuation pattern in the first half of the weekdays is dominated by the cognitive states, while the pattern in the second half is dominated by affective states.

**Figure 2:**
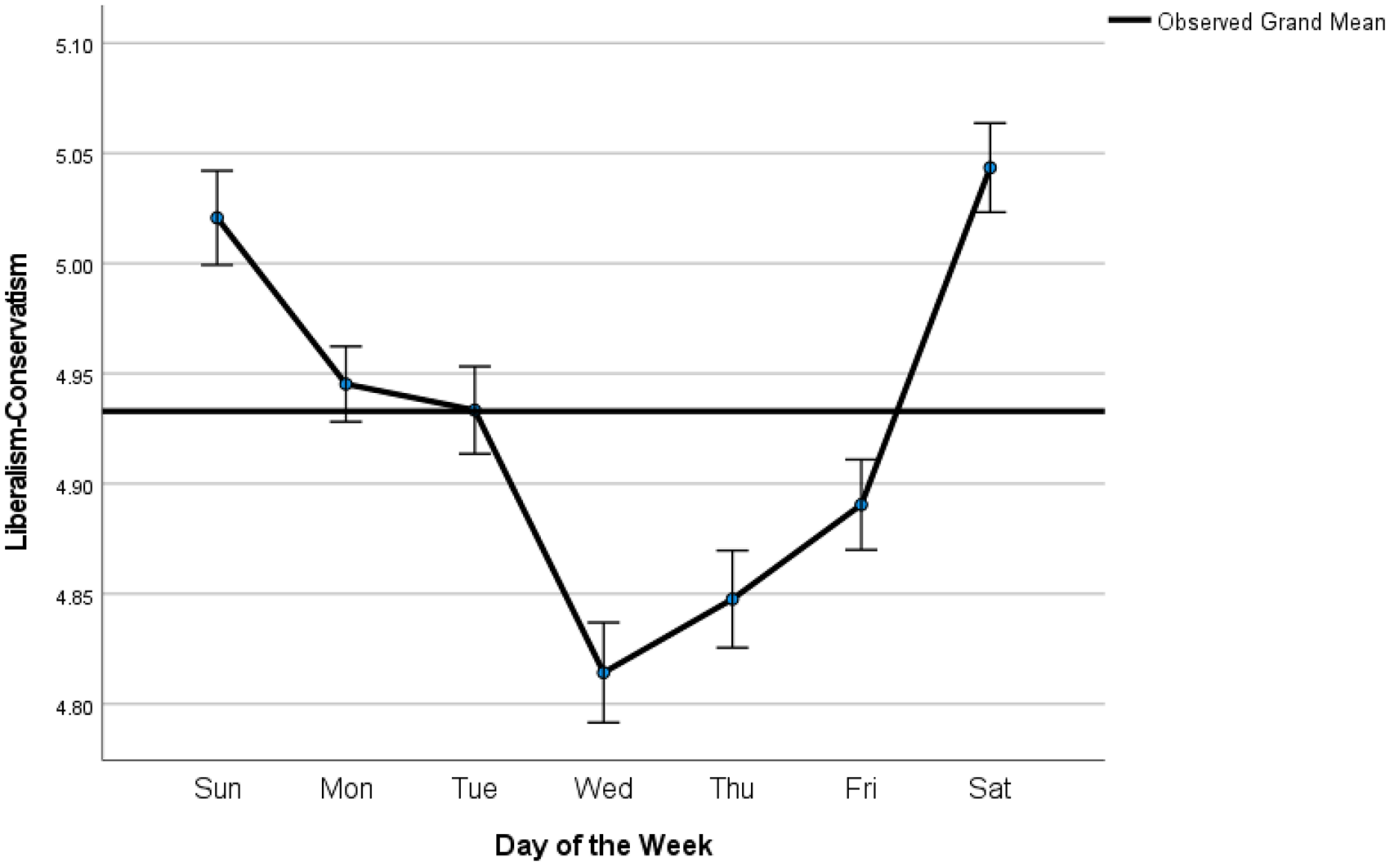
Weekly fluctuation patterns of liberalism-conservatism. The error bars show the 95% CI.

Aside from the overall pattern, a close examination of the difference between specific weekdays also suggests the aggregation of the two effects by cognitive and affective states. For example, a significant decline in liberalism from Sundays and Mondays was observed. This is different from the prediction by cognitive states hypotheses, since cognitive resources tend to be largely preserved on Mondays, which are right after the weekends. On the other hand, the significant drop could be due to affective states, which have been found the most negative on Mondays. Therefore, the aggregation of the two effects could provide most plausible accounts not only for the overall fluctuation pattern, but the specific difference between the weekdays (Figure 2).

The above findings could contribute to the understanding about the nature of ideological thinking, which involve both cognitive and affective processes. According to the dual competition model (Pessoa, 2009), behavior is the outcome of the competition between cognitive and affective processing and their competition can be as deep as competing for executive control resources. Specifically, cognition enables people to pursue their own goals and engage in higher-order processing, whereas affective states may interfere in this process. The degree of interference could depend on the motivations or situations. Previous research has demonstrated that higher-order cognitive processing can suppress affective processing when one is motivated to engage in higher-order reasoning. For instance, it was found that effortful cognitive control could suppress the activity of the amygdala, which is highly relevant to affective processing (Pessoa et al., 2002), like reward-punishment contingency. An EEG study found that effortful cognitive control was associated with increased activation of the dorsal-lateral prefrontal cortex (Harris et al., 2013), a brain region which is highly related to emotional regulation (Nejati et al., 2021). An experimental study using the emotional stop-signal task found that cognitive inhibitory control could disrupt emotional interference, or vice versa, potentially suggesting a two-way connection between higher-order cognitive control and emotions (Kalanthroff et al., 2013). Similarly, Etkin et al. (2006) found that the cingulate cortex in the prefrontal region was associated with the resolution of the affective interference. The higher-level and abstract nature of ideological thinking demands higher cognitive processing and thus affective states become suppressed. Consequently, when cognitive states are positive and relatively preserved during the weekend days and on Mondays, the negative affective states are suppressed and cannot exert much effect, making cognitive states more prominent.

However, as the typical work week continues, cognitive states gradually deteriorate and may fail to suppress the effect of affective states, resulting in the lowest level of liberalism on Wednesdays. When cognitive states continue to weaken in the second half of the week (from Thursdays to weekend days), affective states are becoming more positive and start to strengthen its interference. As a result, there is an increasing trend from Thursdays and thereafter, due to the positive prospect of weekends, as people could forecast their emotions associated with upcoming events (Wilson and Gilbert, 2003). Specifically, people generally experience positive affect and higher subjective well-being during Fridays and the weekends (Ryan et al., 2010; Suk et al., 2021), relative to other weekdays. Individuals have higher levels of positive affect when anticipating the upcoming weekends. Therefore, the second half of the week supports the affective states hypotheses as affective states become more prominent. The substantial increase of the level of liberalism from Fridays to Saturdays further demonstrates the prominence of the affective states in the second half of the week.

Altogether, the findings showed that the DOW effect tended to be resulted from the aggregation of both cognitive and affective processes, instead either one of them. The decrease in liberalism in the first half of the week supported the hypotheses of cognitive states, while its increase in the second half of the week supported the hypotheses of affective states. The level of liberalism peaked at the weekends, providing support to the hypotheses according to both cognitive and affective states.

### Implications of the findings

The DOW effect on liberalism-conservatism can offer insights into human behaviors in different areas. For example, an opinion poll conducted on different days of a week may generate different results (i.e., more conservative during midweeks than weekends) and lead to different decisions in policymaking. Besides, the DOW effect on liberalism-conservatism may contribute to our understanding of decision-making processes such as investment. It was found that liberal individuals would invest more on innovative business, compared with conservatives (Gromet et al., 2013). Moreover, educational programs can be designed in a way that is more consistent with the changes in liberalism. Teaching and learning that focuses on tradition (e.g., culture, history, moral education, etc.) may be scheduled in midweeks when people tend to be more conservative, while activities that demand creativity and openness may be scheduled on other days when liberalism is higher (Dollinger, 2007). Furthermore, the current findings of the DOW effect may offer implications to the 4-Day workweek as aforementioned. Based on the current findings, liberalism tends to trough at the midweek, potentially due to depletion in cognitive resources and decreased positive affect. Hence, it could be possible that, even though cognitive resources start to deplete from the first day of work, a shorter working week may produce more positive affect earlier in the week due to the prospect of non-working days approaching. This may in turn increase liberalism level, potentially benefiting jobs that require creativity and innovativeness. In the long run, it would also be interesting to track the liberalism-conservatism level of those who work 4 days a week to examine if such changes in weekly schedule would result in a different DOW effect.

### Limitations and future research

Despite the significant findings, there are several limitations to be noted. First, the current study utilized cross-sectional self-reported data. This design does not allow us to track the intra-individual changes during the week. Future research may adopt longitudinal design to examine the DOW effect within individuals. Second, the sample predominately consisted of younger populations who had access to the Internet. It would be worthwhile to test the DOW effect among other groups such as the elderly to explore if there are differences in patterns. Besides, sampling issues, such as variability in internet skills and fraudulent survey submissions are common concerns for online surveys (Wright, 2005; Hulland and Miller, 2018), though these effects should be minimal in this study as Pan and Xu (2018) found that results based on this sample were robust and had good generalizability when being validated against other evidence. Third, the online survey did not include direct measures of cognitive and affective states. Future studies may examine if the DOW effect on liberalism-conservatism can be explained by changes in affective and cognitive states. Fourth, the effect sizes of the DOW effect were generally small. Therefore, caution is needed when using the DOW effect to predict the level of liberalism, although small effects are common in big data research (Matz et al., 2017) and useful for a cumulative science (Götz et al., 2021). Fifth, this study did not exclude those who might not fit the general weekly schedules (e.g., people working on weekends) due to non-availability of information. Future research should separate different samples if possible. Sixth, as participants who are more conservative may be less willing to respond to sensitive questions, the current research data might be prone to self-selection bias and/or social desirability bias. Seventh, the survey did not include direct measures of cognitive or affective states. Therefore, further studies are required to examine whether the DOW effect on liberalism-conservatives is due to changes in cognitive or affective states as proposed by the cognitive and affective states hypotheses.

## Data availability statement

The original contributions presented in the study are included in the article/supplementary material, further inquiries can be directed to the corresponding author.

## Ethics statement

Ethical review and approval were not required for the study on human participants in accordance with the local legislation and institutional requirements. Written informed consent from the participants’ legal guardian/next of kin was not required to participate in this study in accordance with the national legislation and the institutional requirements.

## Author contributions

SY conceptualized and analyzed the data. JS and SY wrote the first draft. TN and MM contribute to further revision. All authors contributed to the article and approved the submitted version.

## Conflict of interest

The authors declare that the research was conducted in the absence of any commercial or financial relationships that could be construed as a potential conflict of interest.

## Publisher’s note

All claims expressed in this article are solely those of the authors and do not necessarily represent those of their affiliated organizations, or those of the publisher, the editors and the reviewers. Any product that may be evaluated in this article, or claim that may be made by its manufacturer, is not guaranteed or endorsed by the publisher.

## Footnotes

^1^ https://www.zuobiao.me/
